# Identification of QTL *TGW12* responsible for grain weight in rice based on recombinant inbred line population crossed by wild rice (*Oryza minuta*) introgression line K1561 and *indica* rice G1025

**DOI:** 10.1186/s12863-020-0817-x

**Published:** 2020-02-03

**Authors:** Xiaoqiong Li, Yu Wei, Jun Li, Fangwen Yang, Ying Chen, Yinhua Chen, Sibin Guo, Aihua Sha

**Affiliations:** 1 Rice Research Institute/Guangxi Key Laboratory of Rice Genetics and Breeding, Guangxi Academy of Agricultural Science, Nanning, 530007 People’s Republic of China; 2 Oil Crops Research Institute of Chinese Academy of Agricultural Science, Wuhan, 430062 People’s Republic of China; 3 Hubei Collaborative Innovation Center for Grain Industry, Yangtze University, Jingzhou, People’s Republic of China; 4 Hainan Key Laboratory for Sustainable Utilization of Tropical Bioresource, Hainan University, Haikou, 570228 People’s Republic of China

**Keywords:** Wild rice, Introgression lines, SSR, SLAF, Transcription factor

## Abstract

**Background:**

Limited genetic resource in the cultivated rice may hinder further yield improvement. Some valuable genes that contribute to rice yield may be lost or lacked in the cultivated rice. Identification of the quantitative trait locus (QTL) for yield-related traits such as thousand-grain weight (TGW) from wild rice speices is desired for rice yield improvement.

**Results:**

In this study, sixteen *TGW* QTL were identified from a recombinant inbred line (RIL) population derived from the cross between the introgression line K1561 of *Oryza minuta* and the rice cultivar G1025. *TGW12*, One of most effective QTL was mapped to the region of 204.12 kb between the marker 2,768,345 and marker 2,853,491 of the specific locus amplified fragment (SLAF). The origin of *TGW12* was tested using three markers nearby or within the *TGW12* region, but not clarified yet. Our data indicated thirty-two open reading fragments (ORFs) were present in the region. RT-PCR analysis and sequence alignment showed that the coding domain sequences of *ORF12*, one MADS-box gene, in G1025 and K1561 were different due to alternative slicing, which caused premature transcription termination. The MADS-box gene was considered as a candidate of *TGW12*.

**Conclusion:**

The effective QTL, *TGW12*, was mapped to a segment of 204.12 kb using RILs population and a MADS-box gene was identified among several candidate genes in the segment. The region of *TGW12* should be further narrowed and creation of transgenic lines will reveal the gene function. *TGW12* could be applied for improvement of TGW in breeding program.

## Background

Rice (*Oryza sativa* L.) is the world’s most important cereal crop as a staple food [1]. High rice yield is needed to meet the requirement of rapidly increasing population. Grain weight is important to rice yield, and it is usually represented by thousand-grain weight. Many QTLs for rice grain weight have been mapped to rice chromosomes [2–7]. A dozen of QTLs/genes affecting grain weight have be cloned and functionally charaterized, such as the cytokinin oxidase/dehydrogenase (CKX) *OsCKX2(Gn1a)* [8], the transmembrane protein *GS3* [9, 10 ], and its homolog *DEP1* [11], the RING-type E3 ubiquitin ligase *GW2* (*grain width and weight 2*) [12], the arginine-rich domain nuclear protein *qSW5/GW5* [13], the serine carboxypeptidase *GS5/qTGW5a* [14], the Kelch-like domain *qGL3/ OsPPKL1* [15], the SBP domain transcription factor *GW8 (OsSPL16)* [16], the IAA (indole-3-acetic acid)- glucose hydrolase protein *TGW6* [17], the GNAT-like protein *GW6a* (*Grain weight of chromosome 6*)/*OsglHAT1* [18], the ABC1-like kinase *OsAGSW1* [19], the AP2 transcript factor *qHD5* and *OsSNB* [20, 21], the cytochrome P450 protein *GNS4* [22], the otubain-like protease *WTG1* (*WIDE AND THICK GRAIN 1*) [23], the 16-kDa α-amylase/trypsin inhibitor *RAG2* [24], the GSK3/SHAGGY-Like Kinase *qTGW3* [25]. Some genes such as *GW2, WTG1, OsCKX2, TGW3, TGW6, qSW5/GW5qSW5/GW5, GS3, DEP1, qGL3/ OsPPKL1,*and *OsSNB* negatively regulate grain weight, while others like *GW6a/OsglHAT1, GNS4, GS5/qTGW5a, OsAGSW1, RAG2,* and *GW8 (OsSPL16)* function as positive regulator of grain weight.

To date, QTLs/genes associated with TGW have been mostly cloned from the cultivated rice. However, it is known that the genetic resource of cultivated rice turned quite limited during the process of wild rice domestication, which may hinder the yield improvement of the cultivated rice. Wild rice species should contain many valuable genes that can be used for genetic improvements of cultivated rice [26]. Thus, the genetic resource of wild rice species should be explored and used for rice high-yield breeding. It would be an effective way to widen the genetic basis of cultivated rice by introduction and application of favorable wild rice genes.

*Oryza minuta* (*O. minuta*) is a tetraploid wild rice that possesses a number of favorable yield related genes [27]. Rahman et al. mapped 22 novel yield-related QTLs for 16 agronomic traits using a set of introgression line (IL) of *O. minuta*, and demonstrated that 57% of the QTLs were derived from *O. minuta* [27]*.* In a previous study, we also detected 28 QTLs for yield-related traits using ILs derived from the backcross of IR24 (*O. sativa L*) and *O. minuta*, and found that 46.4% of notable QTLs were from *O. minuta* [28].

To further identify the favorable yield-related genes from *O. minuta*, a recombinant inbred line has been developed by crossing of K1561 to *indica* rice G1025 [28]. K1561 is one out of 192 ILs derived from backcross progenies (BC_4_F_2_) of IR24 and *O. minuta*. It was produced from one time cross of IR24 and *O. minuta,* then four times backcross with IR24 as recurrent parent, and four times self-cross. K1561 shows excellent agronomic traits such as long panicles and high TGW. G1025 is an excellent restorer line that is widely used in Guangxi Province of China with dense grains but light TGW. In this study, QTL mapping was conducted on the advanced RILs population by SSR and SLAF markers. Thirteen QTLs or *TGWs* responsible for TGW were detected under fiver environments, and the most effective QTL *TGW12* was mapped to the segment of 204.12 kb based on the high-density genome map constructed with SLAF. The candidate genes of *TGW12* were preliminarily concluded, and one gene encoding MADS-box protein was considered as the putative candidate based on sequence alignment. The *TGW12* allele for increasing TGW might originated from *O. minuta* and likely used for rice yield improvement.

## Results

### Phenotypic analysis

**Fig. 1:**
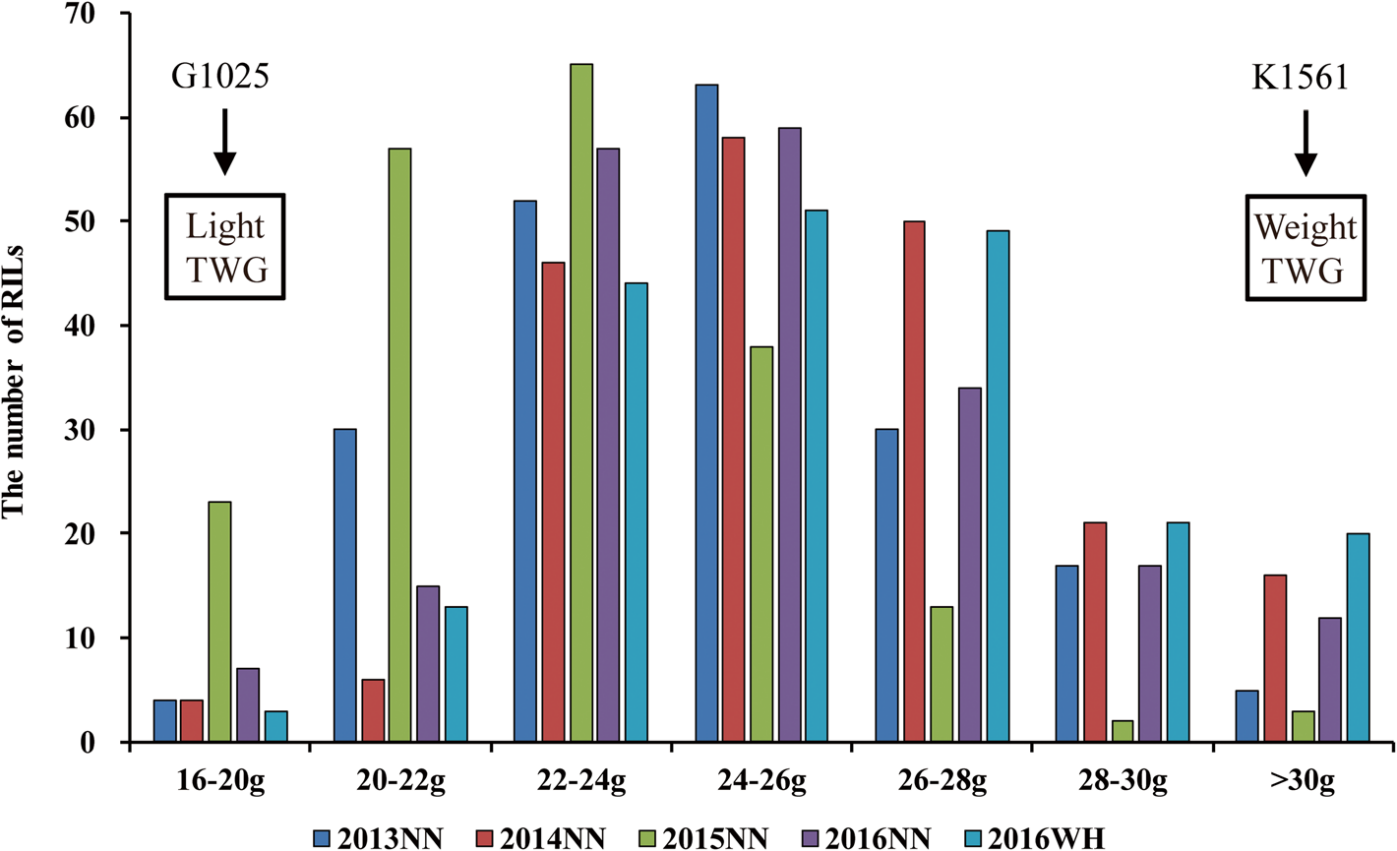
Phenotypic evaluation of thousand-grain weight (TGW) for G1025, K1561 and RILs**.** The Y axis represents the number of RIL lines. The X axis represents the values of TGW: 16 g < TGW ≤ 20 g; 20 < TGW ≤ 22 g; 22 g < TGW ≤ 24 g; 24 g < TGW ≤ 26 g; 26 g < TGW ≤ 28 g; 28 g < TGW ≤ 30 g; TGW > 30 g. G1025 and K1501 are the parents with light and weight TGW, respectively. The arrows indicate the TGW range of the parents (G1025 and K1561), respectively

The two parents G1025 and K1561 showed highly significant differences in TGW under five environments including two locations in Nan-Ning (NN), and Wu-Han (WH), in China with an average of 16.01 g and 32.07 g (Table 1), respectively. TGW values of the 201 individuals were mostly distributed between 20 and 30 g with an average of 24.60 g, 25.69 g, 22.80 g, 24.97 g, and 25.69 g in 2013NN (2013 in Nan-Ning), 2014NN, 2015NN, 2016NN, 2016WH (in 2016 in Wu-Han)(Table 1, Fig. 1, Additional file 1: Table S1), respectively. TGW values of 104, 106, 104, 107, and 107 out of the 201 individuals were smaller than the average in 2013NN, 2014NN, 2015NN, 2016NN, 2016WH, and TGW of the remaining individuals was either equal to or larger than the average (Additional file 1: Table S1).

**Table 1:** Thousand Grain weight of Parental lines G1025, K1561 and RILs

Environments	G1025 (g)	K1561 (g)	RILS	
			Mean(g)	Range(g)
2013NN	16.23 ± 0.07	31.94 ± 0.09	24.60 ± 2.74	16.82–34.67
2014NN	16.89 ± 0.09	32.99 ± 0.12	25.69 ± 2.90	16.68–36.02
2015NN	14.44 ± 0.05	29.58 ± 0.07	22.80 ± 2.48	16.67–31.26
2016NN	15.76 ± 0.06	33.68 ± 0.13	24.97 ± 3.00	15.68–34.05
2016WH	16.73 ± 0.09	32.18 ± 0.11	25.69 ± 3.04	16.69–35.00

*NN* Nanning, *WH* Wuhan

### QTL mapping of *TGW* by simple sequence repeats (SSR)

**Fig. 2:**
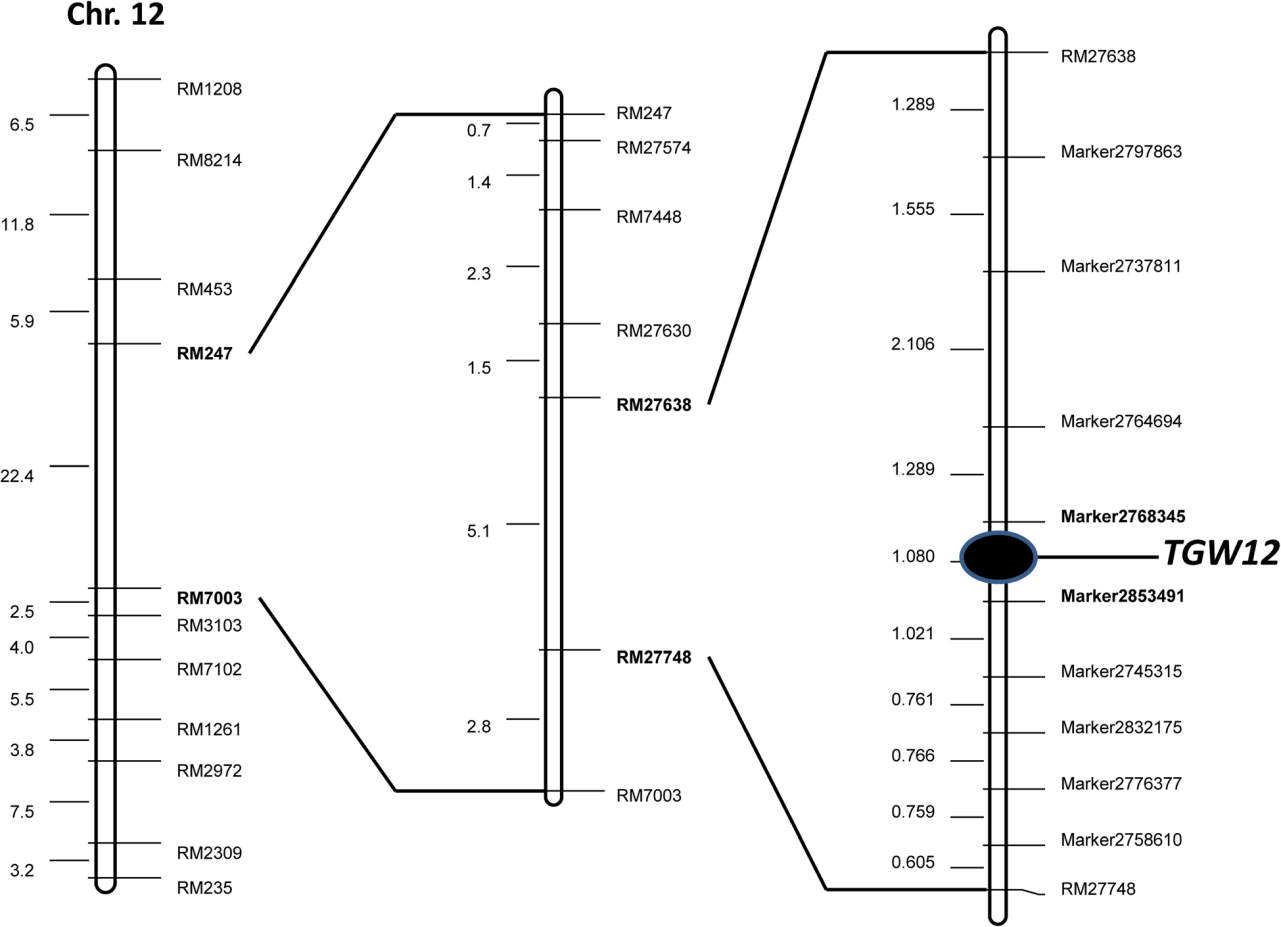
Mapping of *TGW12* by SSR and SLAF Markers

*TGW* QTLs were preliminarily detected by 300 SSR markers evenly distributed on the 12 chromosomes. The population were F_6_, F_7_ RILs derived from the cross of G1025 and K1561 planted in NN in 2013, 2014. Four QTLs *TGW3, TGW7, TGW9.2*, and *TGW12* were stably detected on the chromosomes 3, 7, 9 and 12 in the two environments (Table 2). *TGW12* had the greatest effect, which located on the region of RM247 and RM7003 (Table 2), so it was selected for further analysis. There were other 166 SSR markers (Additional file 2: Table S2) in the region based on the genome sequencing data of *Nipponbare* [29]. The polymorphism of the 166 SSRs was firstly detected between the parental lines G1025 and K1561. As a result, nine SSRs showed polymorphism but only five displayed clear bands. The five SSRs were further used to detect F_6_, F_7_ RILs population in 2013 and 2014. Finally, *TGW12* was mapped to the 5.1 cM region between RM27638 and RM27748 (Fig. 2).

**Table 2:** Quantitative trait loci (QTL) analysis of rice thousand grain weight

Environments	QTL	Chr	Marker interval	Position (cM)	LOD	Additive effect	R^2^ (%)	The donor parent
2013NN	*TGW3*	3	RM186-RM416	95.7	–	0.76	5.83	K1561
	*TGW7*	7	RM455-RM10	76.1	–	1.22	9.25	K1561
	*TGW9.2*	9	RM201-RM6294	85.3	–	1.05	8.07	K1561
	*TGW12*	12	RM7003-RM247	45.6	–	1.93	24.81	K1561
2014NN	*TGW3*	3	RM186-RM416	95.7	–	0.69	4.37	K1561
	*TGW7*	7	RM455-RM10	76.1	–	1.39	10.88	K1561
	*TGW9.2*	9	RM201-RM6294	85.3	–	0.85	5.34	K1561
	*TGW12*	12	RM7003-RM247	45.6	–	1.71	19.12	K1561
2015NN	*TGW7*	7	Marker1254956	99.60	6.94	0.71	8.01	K1561
	*TGW9.1*	9	Marker864241	166.79	9.15	0.84	11.21	K1561
	*TGW12*	12	Marker2768345	56.06	15.42	1.18	22.36	K1561
2016NN	*TGW7*	7	Marker1254956	99.60	7.69	0.99	10.76	K1561
	*TGW9.2*	9	Marker775977	163.15	8.91	1.09	12.95	K1561
	*TGW12*	12	Marker2768345	56.06	11.99	1.26	17.48	K1561
2016WH	*TGW7*	7	Marker1124977	101.91	7.48	0.99	10.43	K1561
	*TGW12*	12	Marker2768345	56.06	11.96	1.30	17.95	K1561

*NN* Nanning, *WH* Wuhan. All the QTL are significant at a level of *P* < 0.01

### QTL mapping of *TGW* by SLAF markers

**Fig. 3:**
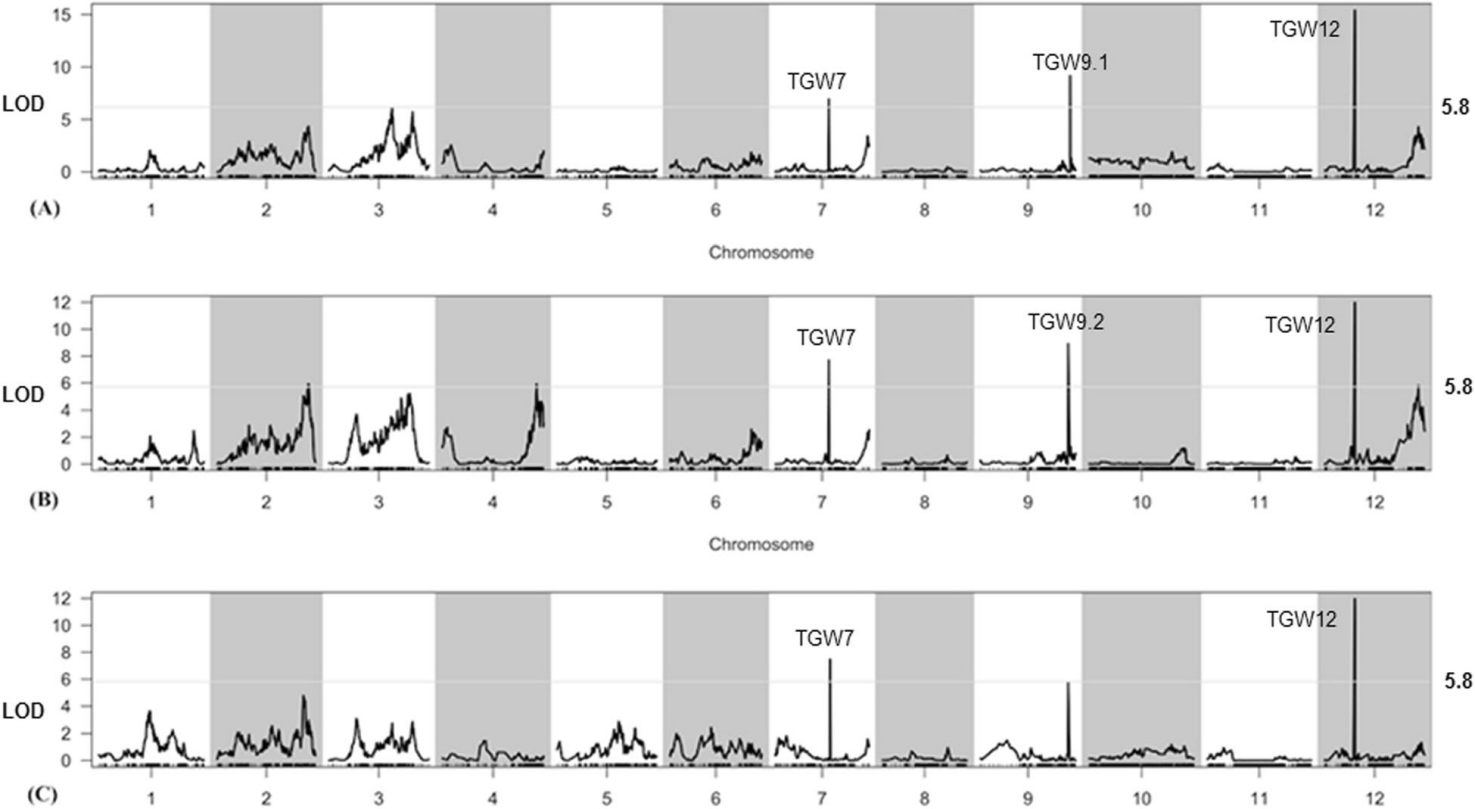
Identification of QTLs for TGW. TGW12 were identified in 2015NN (A), 2016NN (B), and 2016WH (C), which was linked with Marker2768345

We have developed 5521 SLAF markers by SLAF sequencing [30]. To further map *TGW* QTLs, those SLAF markers were used to screen F_8_ RILs in NN in 2015 and F_9_ RILs in NN and WH in 2016, respectively. A total of eight QTLs were detected in three environments, namely, *TGW7, TGW9.1,* and *TGW12* in 2015NN; *TGW7, TGW9.2,* and *TGW12* in 2016NN; *TGW7* and *TGW12* in 2016WH (Table 2, Fig. 3). *TGW7* and *TGW12* were both detected in three environments, and *TGW9.1* and *TGW9.2* were each detected once. *TGW7* explained the phenotypes for 8.01, 10.76, and 10.43% inheritance with an LOD of 6.94, 7.69, and 7.48 in 2015NN, 2016NN, and 2016WH, respectively, whereas *TGW12* showed 22.36, 17.48, and 17.95% inheritance explaining for the phenotypes with an LOD of 15.42, 11.99, and 11.96 in the three environments, respectively (Table 2). *TGW12* had a greater effect than *TGW7*, which was consistent with the results analyzed by SSR mapping (mentioned above). Further analysis for *TGW12* was conducted by comparing the linkage map constructed by SSR and SLAF markers. Consequentially, nine SLAF markers fell into the region of RM27638-RM27748, and *TGW12* was further narrowed to 204.12 kb region between SLAFs Marker 2,768,345 and Marker 2,853,491 (Fig. 2).

### Evaluation of *TGW12* phenotype and genetic origin identification of *TGW12* segment

**Fig. 4:**
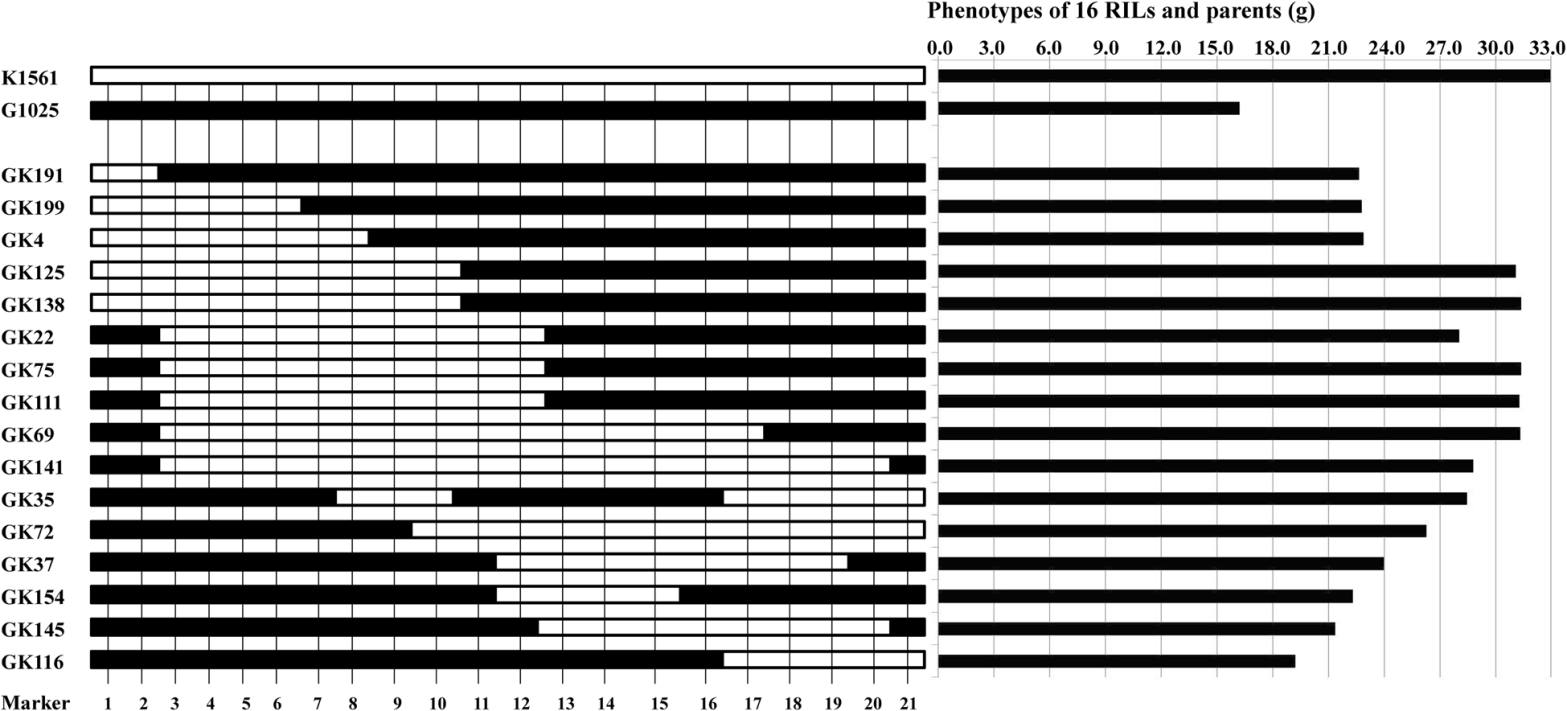
Genotypes and phenotypes of 16 RILs and parents. Left figure, genotypes of 16 RILs and parents. Marker1–21, Marker2804825, Marker2755190, Marker272486, RM27748, Marker2758610, Marker2776377, Marker2832175, Marker2745315, Marker2853491, Marker2768345, Marker2764694, Marker2737811, Marker2797863, RM27638, Marker2734798, Marker2854577, Marker2827288, Marker2753963, Marker2716467, Marker2852418, Marker2816785, respectively. Solid bar represents segments of G1025, and hallow bar represents segments of K1561. Right figure, phenotypes of 13 RILs and parents

**Fig. 5:**
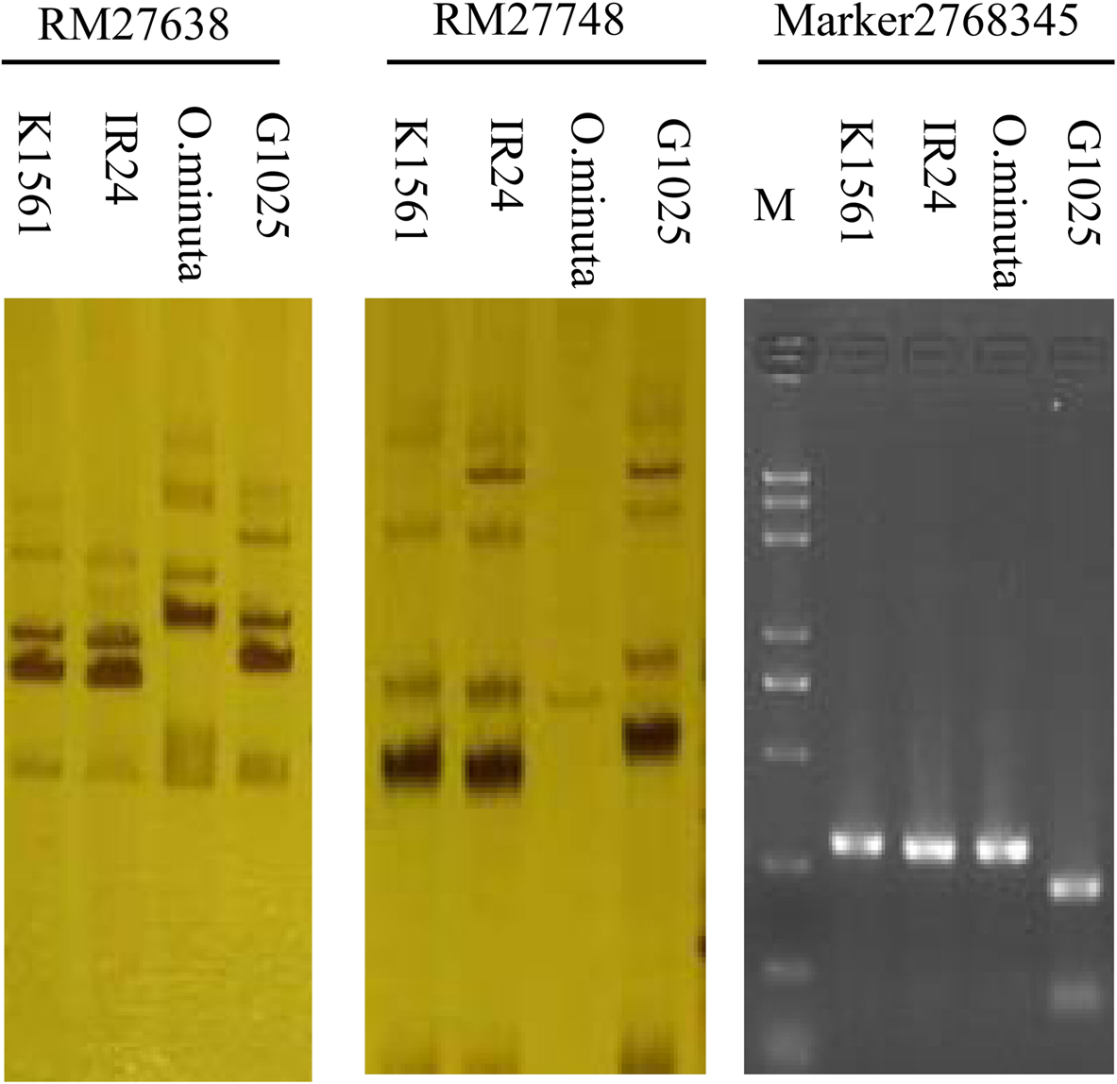
Identification of *TGW12* segment by SSR or SLAF markers. Lanes1–4, K1561, IR24, *O. minuta*, G1025. M, DNA marker 2000 plus

In order to evaluate whether the phenotypes were determined by *TGW12*, 16 out of the 201 RILs containing the *TGW12* region were identified by means of the markers nearby the region (Fig. 4). Then, the phenotypes and genotypes of the 16 RILs were compared. All the 16 RILs with one or two segments of K1561 showed TGW increase than the recurrent parent G1025, suggesting *TGW12* control TGW (Fig. 4). To clarify whether the increasing effect of *TGW12* was originated from *O. minuta,* the genotypes of G1025, K1561, IR24, and *O. minuta* were examined using markers nearby or within *TGW12*. The genotype of K1561 was the same as that of IR24 but different from that of G1025 and *O. minuta* on the sites of RM27638 and RM27748, which are nearby *TGW12* (Figs. 2 and 5). However, the genotype of K1561 was the same as those of IR24 and *O. minuta,* but it was different from that of G1025 at the site of Marker 2,768,345, which is linked with *TGW12*. It was hard to draw a conclusion whether *TGW12* originated from IR24 or *O. minuta* based on the above results*.* It has been suggested that translocation through centric break-fusion occurred more frequently than recombination in the introgression lines with interspecific cross, which didn’t always result in an *O. minuta* chromosome arm onto a complete or incomplete *O. sativa* chromosome [30, 31]. Thus, *TGW12* origination remains to be determined in the near future. It is feasible to compare sequence of *TGW12* candidate among *O. minuta,* IR24, and K1561 once it was fine mapped.

### Preliminary prediction of candidate genes for *TGW12*

**Fig. 6:**
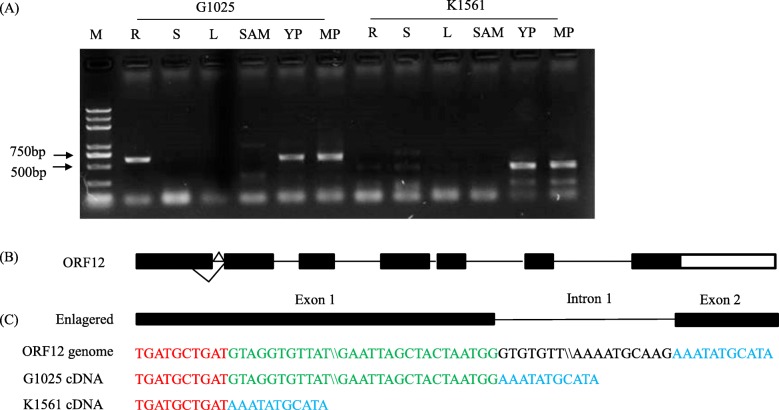
RT-PCR for ORF12 and sequence alignment. **a** RT-PCR for *ORF12*. M, DNA marker 2000 plus; R, root; S, stem; L, leave; SAM, shoot apical meristem; YP, young panicles (1–5 cm); MP, mature panicles (15–20 cm). **b** Genomic structure of *ORF12.* Solid bar, exon; hollow bar, 3’untranslated region; line, intron; fold lines, alternative splicing. **c** the cDNA sequence alignment of *ORF12* between G1025 and K1561. The red and green letters indicated the sequence of exon1, and the sequence showed in green letters are those kept in the cDNA of G1025 but spliced in K1561; the blue letters indicated the sequence of exon2; the black letters indicated the sequence of intron. The symbol of “\\” indicated the omitted sequences

Analysis of annotated genes indicated that 32 *ORFs* located in the 204.12 kb region based on *Nipponbare* genome annotation (http://rice.plantbiology.msu.edu) (Table 3). Among them, 13 ORFs encoded functional proteins and 19 *ORFs* were annotated as transposon/retrotransposon proteins, hypothetical proteins, or expressed protein. It is worth noting that there were four transcription factors (TFs) among the functional proteins: two MADS-box proteins (*ORF12*, *ORF14*), one ZF-HD protein (*ORF24*), and one B-box zinc finger protein (*ORF27*). Because TFs play crucial roles in regulation of plant growth and development [32], the four TFs were considered preferentially as putative candidate genes of *TGW12*. Reverse transcript (RT)-PCR were conducted to amplify the CDS (coding domain sequence) of the four transcription factors from the parents G1025 and K1561. Sequence comparison indicated that the amplified sequence of *ORF12* in K1561 was 56 bp shorter than that of G1025, which resulted in premature transcription termination, consequently leading to a peptide with 45 amino acid residues only in K1561, whereas *ORF12* in G1025 encoded a protein with 202 amino acid residues (Additional file 3: Figure S1). Further analysis revealed that lack of 56 bp of *ORF12* in K1561 was due to alternative splicing (AS) in the first extron (Fig. 6b, c), which causes premature termination of ORF12 translation (Additional file 3: Figure S1). There were no difference in the CDS of *ORF14*, *ORF24*, and *ORF27* between K1561 and G1025 (data not shown). Thus, the *MADS-box* (*ORF12*) was likely one putative candidate of *TGW12*. However, possibility of other nine functional proteins and the hypothetical proteins, or expressed protein independently or collectively affecting TGW could not be excluded. Further investigation is required.

**Table 3:** Predicted candidate genes of TGW12

ORFs	Gene product	ORFs	Gene product
ORF1	NB-ARC/LRR disease resistance protein	ORF17	ATP synthase subunit beta
ORF2	Ty3-gypsy retrotransposon protein	ORF18	Ribulose bisphosphate carboxylase large chain precursor
ORF3	Ty3-gypsy retrotransposon protein	ORF19	OsClp13 - Putative Clp protease homologue
ORF4	Ty3-gypsy retrotransposon protein	ORF20	Photosystem II P680 chlorophyll A apoprotein
ORF5	expressed protein	ORF21	Retrotransposon protein
ORF6	rp1	ORF22	Ty3-gypsy retrotransposon protein
ORF7	expressed protein	ORF23	Ty3-gypsy retrotransposon protein
ORF8	hypothetical protein	ORF24	ZF-HD protein
ORF9	expressed protein	ORF25	uncharacterized protein ycf45
ORF10	Ty1-copia retrotransposon protein	ORF26	expressed protein
ORF11	expressed protein	ORF27	B-box zinc finger family protein
ORF12	MADS-box family gene	ORF28	AAA-type ATPase family protein
ORF13	expressed protein	ORF29	Retrotransposon protein
ORF14	MADS-box family gene	ORF30	expressed protein
ORF15	CACTA transposon protein	ORF31	expressed protein
ORF16	Clathrin adaptor complex small chain domain containing protein	ORF32	NB-ARC domain containing protein

## Discussion

Rice is one of the most important staple food widely consumed by one-half world’s peopulation and more product is needed as population increases in future. However, further yield improvement of rice is constrained by the narrow genetic basis of cultivated rice varieties. Wild rice species are good candidates to explore the genetic resource for valuably genes to further enhance rice productivity [26].

*O. minuta* possesses a number of outstanding genes associated with resistance and yield [27]. Lots of QTLs for yield related traits have been identified using the introgression lines (IL) consisting of *O. minuta* segments [27, 28]. In this study, sixteen *TGWs* were detected using the advanced RILs population under five environments (Table 2). Among them, the most effective QTL, *TGW12*, was mapped on chromosome 12. Eight QTLs for TGW (AQAG040, AQCF014, AQAG053, AQGP079, AQDR045, AQDR047, AQCY020, CQAS153) had been previously mapped to Chromosome 12 (http://archive.gramene.org/). Location comparison indicated that *TGW12* was partially overlapped with AQDR045. *TGW12* and AQDR045 were respectively located in the regions of 4,037,811–6,150,143 and 1,589,200–5,829,185 on Chromosome 12 in the physical map of *Nipponbare* genome [29], suggesting that there might be a major QTL controlling grain weigh in this region. However, AQDR045 was mapped by using the popu1ation derived from two cultivated rice Lemont and Teqing [33], whereas *TGW12* was mapped using the population of one *O. minuta* introgression line and one cultivated line. Although it was uncertain whether *TGW12* allele originated from *O. minuta* or IR24, it showed a great increasing effect for TGW and could be directly applied in the breeding program.

There were 32 annotated *ORFs* in the *TGW12* region, among which four *ORFs* were identified as TFs based on sequence alignment and considered as *TGW12* candidates due to their regulatory roles in plant growth and development. Sequence analysis indicated that *ORF12* in K1561 was truncated, with 45 amino acid residues only, due to an AS event while the counterpart in G1025 possessed a full length of the protein. No sequence difference in the other three TFs (ORF14, ORF24, and ORF27) was found between K1561 and G1025. *ORF12* encoded a MADS-box protein, which possesses a highly conserved DNA-binding MADS domain and is involved predominantly in developmental processes [34]. In *Arabidopsis*, there are 107 genes encoding MADS-box proteins [34], and almost all of them are involved in the process of flower and seed development [35]. In rice, 75 *MADS-box* genes were identified, and more than 20 were transcribed during the stages of panicle and seed development [36]. In addition, alternative splicing of one MADS-box transcription factor *OsMADS1* encoded by *OsLG3b (Os03g0215400)* controls grain length and yield in *japonica* rice [37]. Our results suggested that *ORF12* could be critical for the function of *TGW12*, even functioned as *TGW12.* However, further studies are required to examine the function of other ORFs located in the region.

## Conclusions

In this study, an effective QTL *TGW12* related to the trait of thousand-grain weight in rice was mapped to a segment with 204.12 kb using RILs population derived from the cross progenies of one *O. minuta* introgression line and one cultivated rice. Out of 32 *ORFs* located in the region of *TGW12*, ORF12 encoded a MADS-box protein could be crucial for the *TGW12* function. Further investigation is required to validate this speculation.

## Methods

### Plant materials and field trials

*O. minuta* (Acc. No. 101133) and IR24 were kindly provided by the International Rice Germplasm Centre of the International Rice Research Institute. The parental line G1025 was kindly provide by Rice Research Institute of Guangxi Academy of Agricultural Science. The parental lines K1561 was developed by our lab in Rice Research Institute of Guangxi Academy of Agricultural Science. It is produced by one time cross of IR24 and *O. minuta,* then four times backcross with IR24 as recurrent parent, and four times self-cross. The parental lines G1025 and K1561 along with 201 F_6_, F_7_ RILs were planted in Nanning (NN) from February to July in 2013 and 2014, respectively. The parental lines along with 201 F_8_ RILs were planted in NN (February to July) in 2015, and parents and F_9_ RILs were planted in NN (February to July) and Wuhan (WH) from May to October in 2016, respectively. The phenotypes of parents and RILs were collected to map TGW based on SSR or SLAFs. Grain weight was calculated based on 200 grains and converted to TGW after harvesting and sun-drying. The mean values of ten plants were used as input data to identify QTLs (Additional file 1: Table S1).

### SSR, linkage, and QTL analysis

DNA was extracted from fresh leaves following the CTAB procedure [38]. SSR markers were used to analyze a polymorphism between the parents (Additional file 2: Table S2). SSR were synthesized according to published sequences [29]. Polymerase chain reaction (PCR) was conducted in a 15 μL volume as follow: 50 ng of template DNA, 0.3 μL of 10 mM each dNTPs, 0.5 units of Taq DNA polymerase, 1.5 μL of 10 × PCR buffer with Mg^2+^, and 0.5 μL of 10 μM forward and reverse primers. The reaction conditions was carried out as an initial denaturation at 94 °C for 5 min, followed by 35 cycles of 30 s at 94 °C, 30 s at 56 °C, and 30 s at 72 °C, with a final extension at 72 °C for 10 min. PCR products were separated on 6% polyacrylamide denaturing gels, and the bands were revealed by the silver-staining protocol [39].

Linkage was constructed by Mapmaker/Exp 3.0 [40]. Genetic distance was calculated by the Kosambi function. QTLNetwork2.2 was used to analyze QTL at a threshold of LOD 3.0 [41].

### Single nucleotide polymorphism (SNP) genotyping, linkage map construction and QTL analysis

Genomic DNA was extracted from fresh leaves of the parents and RILs by CTAB [38]. Quantified DNA was used for SLAF sequencing by an Illumina HiseqTM 2500 [42]. SLAF markers, developed in previous work, were used for genotyping, linkage map construction and QTL analysis for TGW in this study as described by Zhu et al. [30].

### Derived cleaved amplified polymorphic sequences (dCAPS) marker development

dCAPS marker was developed for SLAF Marker2768345. Primers were designed according to dCAPS Finder 2.0 (http://helix.wustl.edu/dcaps/dcaps.html). PCR were conducted in a 20 μL volume as follow: 100 ng of template DNA, 0.5 μL of 10 mM each dNTPs, 1 units of Taq DNA polymerase, 2.0 μL of 10 × PCR buffer with Mg^2+^, and 0.5 μL of 10 μM forward and reverse primers. The reaction conditions was carried out as an initial denaturation at 94 °C for 3 min, followed by 35 cycles of 30 s at 94 °C, 30 s at 60 °C, and 30 s at 72 °C, with a final extension at 72 °C for 5 min. PCR products were digested with *Eco*R V (Takara, China) for 4 h at 37 °C, then were resolved in 2% agrose gel to genotype.

### Candidate gene prediction and RT-PCR analysis

The predicted genes in the target region of QTL were analyzed according to the annotation of Nipponbare reference genome2. RT-PCR was conducted as described by Sha et al. [43]. In brief, total RNA was extracted from roots, leaves, stems, shoot apical meristem, young panicles (1–5 cm), mature panicles (15–20 cm) of K1561 and G1025 with Trizol (Invitrogen, Carlsbad, CA, USA). RNAs were digested with DNase I (Promega, USA) to eliminate genomic DNA contamination before cDNA synthesis. For cDNA synthesis, 5.0 μg total RNA was used for reverse transcribed by M-MLV Reverse Transcriptase (Promega, USA) using oligo-d (T) according to the user manual. For RT-PCR analysis, 2 μl of the first cDNA strand was used with gene-specific primer pairs in 20 μL reaction volumes, which contained 1 μM forward and reverse primer each, 200 μM dNTPs, and 1 U Taq enzyme (Takara, China). A DNA Engine peltier ThermalCycler (Bio-RAD) was used to perform the reaction. The PCR procedure was as follows: 94 °C for 2 min; 35 cycles of 94 °C for 30 s, 53 °C for 30 s, 72 °C for 1 min per 1 kb; 5 min for a final elongation at 72 °C. The PCR products were purified from agarose gel after electrophoresis and cloned into pMD-18 T vector (Takara, China). Clones were sequenced by Tianyihuiyuan Company (Wuhan, China). Sequences were aligned by ClustalW (https://www.genome.jp/tools-bin/clustalw).

## Supplementary information

**Additional file 1:** **Table S1**. Phenotypes of parents and RILs in different environments.

**Additional file 2:** **Table S2**. SSR primer sequences used in the present study.

**Additional file 3:** **Figure S1**. Alignment of amino acid sequences of ORF12 between G1025 and K1561.

## Data Availability

The datasets used and/or analyzed during the current study are available from the corresponding author on reasonable request.

## Abbreviations

CKX: Cytokinin oxidase/dehydrogenase
dCAPS: Derived cleaved amplified polymorphic sequences
GW2: Grain width and weight 2
GW6a: Grain weight of chromosome 6
IAA: Indole-3-acetic acid
ORFs: Open reading fragments
PCR: Polymerase chain reaction
QTL: Quantitative trait locus
RIL: Recombinant inbred line
RT-PCR: Reverse transcript (RT)-PCR
SLAF: Specific locus amplified fragment
SNP: Single nucleotide polymorphism
SSR: Simple sequence repeats
TGW: Thousand-grain weight
WTG1: Wide and thick grain 1
