# Supplementary material for: Identification of QTL TGW12 responsible for grain weight in rice based on recombinant inbred line population crossed by wild rice (Oryza minuta) introgression line K1561 and indica rice G1025
Source: BMC Genet. 2020 Feb 3;21:10. doi: 10.1186/s12863-020-0817-x (PMC6998338; doi:10.1186/s12863-020-0817-x)
Supplement: Supplementary file 1 — Additional file 1: Table S1. Phenotypes of parents and RILs in different environments. [file 12863_2020_817_MOESM1_ESM.docx]

Table S1 Phenotype of parental lines K1561, G1025 and RILs

| RILs/parents | 2013NN | 2014NN | 2015NN | 2016NN | 2016WH |
| --- | --- | --- | --- | --- | --- |
| GX1 | 18.36 | 19.65 | 17.55 | 20.14 | 20.31 |
| GX2 | 22.90 | 24.33 | 21.63 | 24.50 | 27.05 |
| GX3 | 23.88 | 24.76 | 23.20 | 24.95 | 26.20 |
| GX4 | 23.14 | 23.53 | 20.92 | 21.70 | 24.05 |
| GX5 | 23.19 | 24.86 | 21.68 | 25.02 | 27.57 |
| GX6 | 23.73 | 25.45 | 22.00 | 25.43 | 27.93 |
| GX7 | 25.65 | 27.75 | 24.52 | 28.71 | 27.37 |
| GX8 | 26.12 | 26.23 | 21.86 | 22.07 | 24.14 |
| GX9 | 23.49 | 24.41 | 24.61 | 26.46 | 28.76 |
| GX10 | 24.80 | 26.67 | 21.64 | 25.38 | 24.96 |
| GX11 | 22.34 | 23.39 | 20.38 | 22.48 | 23.65 |
| GX12 | 28.25 | 28.66 | 26.38 | 27.21 | 26.58 |
| GX13 | 23.18 | 23.60 | 22.36 | 23.21 | 22.00 |
| GX14 | 26.97 | 28.69 | 23.62 | 27.07 | 27.81 |
| GX15 | 25.47 | 26.98 | 24.22 | 27.24 | 25.31 |
| GX16 | 22.84 | 23.38 | 21.22 | 22.29 | 23.53 |
| GX17 | 24.06 | 25.68 | 22.08 | 25.32 | 22.13 |
| GX18 | 25.91 | 26.21 | 23.90 | 24.49 | 25.10 |
| GX19 | 28.54 | 28.46 | 26.21 | 26.04 | 27.02 |
| GX20 | 21.28 | 22.56 | 19.70 | 22.26 | 22.49 |
| GX21 | 24.08 | 25.42 | 22.19 | 24.86 | 23.65 |
| GX22 | 28.71 | 29.19 | 26.96 | 27.91 | 28.19 |
| GX23 | 21.87 | 24.39 | 20.00 | 25.03 | 24.90 |
| GX24 | 22.68 | 24.21 | 20.99 | 24.05 | 21.53 |
| GX25 | 25.82 | 28.49 | 23.93 | 29.26 | 25.56 |
| GX26 | 27.82 | 29.19 | 25.14 | 27.88 | 28.07 |
| GX27 | 25.37 | 27.42 | 22.94 | 27.05 | 26.07 |
| GX28 | 23.04 | 23.46 | 21.92 | 22.77 | 25.10 |
| GX29 | 28.77 | 29.68 | 27.16 | 28.99 | 28.79 |
| GX30 | 23.72 | 25.85 | 23.35 | 27.60 | 27.67 |
| GX31 | 24.03 | 24.72 | 22.27 | 23.65 | 23.74 |
| GX32 | 27.77 | 28.09 | 22.71 | 23.36 | 22.26 |
| GX33 | 29.43 | 29.76 | 27.66 | 28.33 | 30.42 |
| GX34 | 24.43 | 26.85 | 24.37 | 29.21 | 25.68 |
| GX35 | 25.17 | 26.63 | 25.99 | 28.91 | 28.00 |
| GX36 | 24.36 | 25.60 | 19.91 | 22.39 | 25.74 |
| GX37 | 26.21 | 26.63 | 22.51 | 23.36 | 24.63 |
| GX38 | 28.07 | 28.50 | 25.00 | 25.86 | 26.92 |
| GX39 | 25.16 | 27.38 | 24.70 | 29.13 | 27.57 |
| GX40 | 24.29 | 24.68 | 23.19 | 23.98 | 24.09 |
| GX41 | 21.65 | 22.85 | 21.35 | 23.76 | 24.33 |
| GX42 | 23.13 | 24.19 | 21.78 | 23.90 | 26.10 |
| GX43 | 24.54 | 27.09 | 20.92 | 26.01 | 25.59 |
| GX44 | 23.28 | 23.91 | 22.34 | 23.60 | 25.15 |
| GX45 | 24.11 | 24.52 | 22.12 | 22.95 | 22.99 |
| GX46 | 21.17 | 22.34 | 20.00 | 22.34 | 22.43 |
| GX47 | 16.82 | 16.68 | 16.67 | 16.40 | 16.69 |
| GX48 | 23.69 | 24.26 | 21.07 | 22.21 | 25.06 |
| GX49 | 22.93 | 23.87 | 22.48 | 24.36 | 23.36 |
| GX50 | 22.49 | 22.64 | 22.56 | 22.86 | 28.38 |
| GX51 | 20.62 | 20.79 | 20.41 | 20.75 | 21.91 |
| GX52 | 27.43 | 30.39 | 23.05 | 28.96 | 26.57 |
| GX53 | 20.49 | 22.76 | 18.29 | 22.82 | 23.01 |
| GX54 | 20.80 | 22.58 | 19.07 | 22.62 | 26.08 |
| GX55 | 21.86 | 23.54 | 20.82 | 24.17 | 22.18 |
| GX56 | 22.90 | 24.12 | 21.98 | 24.42 | 23.27 |
| GX57 | 23.08 | 26.25 | 18.70 | 25.03 | 26.56 |
| GX58 | 24.36 | 26.50 | 21.01 | 25.28 | 30.74 |
| GX59 | 23.83 | 24.25 | 23.15 | 24.00 | 24.47 |
| GX60 | 23.07 | 24.94 | 22.29 | 26.01 | 29.85 |
| GX61 | 24.89 | 26.45 | 21.81 | 24.92 | 24.07 |
| GX62 | 20.44 | 22.09 | 19.25 | 22.54 | 35.00 |
| GX63 | 26.94 | 27.39 | 24.65 | 25.56 | 27.89 |
| GX64 | 25.31 | 26.19 | 23.31 | 25.08 | 28.40 |
| GX65 | 27.75 | 28.76 | 25.71 | 27.73 | 29.42 |
| GX66 | 24.31 | 26.36 | 22.82 | 26.91 | 30.94 |
| GX67 | 22.13 | 23.04 | 20.99 | 22.80 | 23.68 |
| GX68 | 21.12 | 22.95 | 18.77 | 22.43 | 22.82 |
| GX69 | 28.09 | 30.59 | 26.01 | 31.01 | 31.69 |
| GX70 | 24.85 | 25.85 | 23.28 | 25.28 | 30.40 |
| GX71 | 20.29 | 20.69 | 19.16 | 19.95 | 21.32 |
| GX72 | 22.31 | 23.33 | 23.11 | 25.15 | 27.41 |
| GX73 | 21.03 | 22.33 | 20.33 | 22.92 | 23.82 |
| GX74 | 21.88 | 22.21 | 21.43 | 22.09 | 30.06 |
| GX75 | 27.77 | 30.76 | 25.79 | 31.76 | 30.97 |
| GX76 | 20.61 | 22.51 | 19.82 | 23.63 | 22.71 |
| GX77 | 20.87 | 22.66 | 18.88 | 22.47 | 21.67 |
| GX78 | 20.60 | 21.23 | 19.15 | 20.41 | 23.14 |
| GX79 | 25.72 | 26.01 | 23.93 | 24.50 | 29.59 |
| GX80 | 22.50 | 23.23 | 20.26 | 21.73 | 24.32 |
| GX81 | 26.34 | 23.82 | 24.35 | 19.32 | 30.37 |
| GX82 | 21.96 | 24.17 | 19.94 | 24.36 | 23.76 |
| GX83 | 27.57 | 29.59 | 23.49 | 27.51 | 28.41 |
| GX84 | 21.65 | 21.98 | 20.88 | 21.54 | 23.40 |
| GX85 | 18.80 | 18.84 | 18.32 | 18.40 | 20.83 |
| GX86 | 20.60 | 22.39 | 18.98 | 22.57 | 21.93 |
| GX87 | 22.92 | 23.15 | 21.51 | 21.98 | 34.01 |
| GX88 | 25.86 | 27.99 | 24.17 | 28.42 | 25.27 |
| GX89 | 23.17 | 24.93 | 20.80 | 24.32 | 24.30 |
| GX90 | 24.11 | 24.85 | 21.38 | 22.86 | 24.24 |
| GX91 | 26.03 | 27.00 | 22.80 | 24.74 | 25.81 |
| GX92 | 25.52 | 26.59 | 24.95 | 27.10 | 27.22 |
| GX93 | 23.99 | 24.90 | 22.05 | 23.87 | 24.35 |
| GX94 | 21.71 | 23.24 | 20.69 | 23.73 | 24.10 |
| GX95 | 23.40 | 25.26 | 21.99 | 25.70 | 23.20 |
| GX96 | 24.75 | 25.96 | 23.37 | 25.79 | 26.07 |
| GX97 | 25.10 | 25.70 | 23.35 | 24.55 | 25.78 |
| GX98 | 21.54 | 23.22 | 20.17 | 23.52 | 23.38 |
| GX99 | 25.33 | 27.34 | 22.64 | 26.67 | 26.22 |
| GX100 | 25.26 | 27.86 | 22.80 | 28.00 | 28.14 |
| GX101 | 22.50 | 25.04 | 20.09 | 25.17 | 24.67 |
| GX102 | 27.33 | 27.16 | 25.64 | 25.29 | 28.39 |
| GX103 | 25.88 | 28.26 | 22.94 | 27.70 | 28.09 |
| GX104 | 28.32 | 28.72 | 26.82 | 27.62 | 28.59 |
| GX105 | 32.95 | 34.12 | 30.53 | 32.87 | 34.03 |
| GX106 | 28.37 | 30.33 | 25.81 | 29.74 | 30.00 |
| GX107 | 24.27 | 25.58 | 20.89 | 23.51 | 23.55 |
| GX108 | 25.42 | 26.66 | 23.66 | 26.14 | 26.58 |
| GX109 | 25.08 | 25.56 | 23.12 | 24.07 | 26.34 |
| GX110 | 26.45 | 26.77 | 25.00 | 25.63 | 27.03 |
| GX111 | 28.79 | 31.78 | 25.99 | 31.98 | 30.57 |
| GX112 | 24.96 | 26.13 | 22.79 | 25.13 | 27.90 |
| GX113 | 24.68 | 25.02 | 22.36 | 23.04 | 25.37 |
| GX114 | 25.87 | 26.52 | 24.52 | 25.83 | 26.46 |
| GX115 | 25.10 | 26.88 | 23.12 | 26.67 | 24.49 |
| GX116 | 18.96 | 19.00 | 18.71 | 18.79 | 19.67 |
| GX117 | 24.20 | 25.34 | 23.24 | 25.53 | 24.81 |
| GX118 | 26.25 | 26.29 | 25.35 | 25.44 | 30.00 |
| GX119 | 26.59 | 27.23 | 25.31 | 26.59 | 26.93 |
| GX120 | 23.83 | 23.92 | 21.82 | 22.00 | 25.61 |
| GX121 | 23.66 | 23.26 | 21.93 | 21.12 | 23.53 |
| GX122 | 22.02 | 22.61 | 21.67 | 22.85 | 22.25 |
| GX123 | 22.67 | 23.68 | 21.15 | 23.16 | 23.52 |
| GX124 | 25.04 | 26.02 | 23.45 | 25.41 | 24.71 |
| GX125 | 28.88 | 31.92 | 26.82 | 32.88 | 29.33 |
| GX126 | 27.79 | 29.40 | 25.30 | 28.53 | 27.66 |
| GX127 | 26.42 | 26.86 | 25.71 | 26.61 | 26.27 |
| GX128 | 24.90 | 24.37 | 23.88 | 22.80 | 25.11 |
| GX129 | 24.07 | 24.06 | 23.23 | 23.21 | 24.40 |
| GX130 | 22.41 | 24.20 | 21.63 | 25.23 | 24.08 |
| GX131 | 27.39 | 29.69 | 25.56 | 30.15 | 25.57 |
| GX132 | 22.84 | 22.32 | 16.72 | 15.68 | 17.53 |
| GX133 | 27.11 | 28.26 | 24.72 | 27.02 | 27.76 |
| GX134 | 26.74 | 27.16 | 25.36 | 26.18 | 26.68 |
| GX135 | 26.77 | 26.17 | 26.16 | 24.97 | 25.97 |
| GX136 | 23.88 | 24.62 | 23.60 | 25.08 | 26.06 |
| GX137 | 27.64 | 28.82 | 25.50 | 27.87 | 27.58 |
| GX138 | 29.86 | 32.39 | 29.00 | 34.05 | 28.75 |
| GX139 | 22.98 | 23.98 | 21.15 | 23.13 | 24.76 |
| GX140 | 24.27 | 26.35 | 24.14 | 28.30 | 24.01 |
| GX141 | 28.77 | 31.03 | 26.56 | 31.09 | 26.57 |
| GX142 | 25.89 | 25.48 | 24.33 | 23.52 | 28.45 |
| GX143 | 24.54 | 25.78 | 22.03 | 24.51 | 26.37 |
| GX144 | 23.60 | 24.39 | 22.80 | 24.37 | 23.96 |
| GX145 | 21.17 | 21.95 | 19.68 | 21.23 | 21.50 |
| GX146 | 21.39 | 22.54 | 19.81 | 22.11 | 20.53 |
| GX147 | 34.67 | 36.02 | 30.59 | 33.29 | 32.19 |
| GX148 | 21.83 | 23.42 | 20.29 | 23.46 | 23.23 |
| GX149 | 23.70 | 24.67 | 21.88 | 23.82 | 23.62 |
| GX150 | 28.37 | 28.59 | 26.88 | 27.30 | 27.49 |
| GX151 | 23.37 | 24.75 | 21.74 | 24.50 | 23.61 |
| GX152 | 24.60 | 26.08 | 23.14 | 26.11 | 24.72 |
| GX153 | 24.98 | 26.96 | 23.30 | 27.26 | 26.74 |
| GX154 | 22.23 | 22.99 | 20.63 | 22.14 | 22.49 |
| GX155 | 24.66 | 24.10 | 21.93 | 20.81 | 22.34 |
| GX156 | 28.83 | 30.71 | 25.72 | 29.48 | 30.37 |
| GX157 | 22.85 | 23.28 | 21.75 | 22.61 | 26.06 |
| GX158 | 22.44 | 23.88 | 21.05 | 23.94 | 22.14 |
| GX159 | 24.70 | 25.28 | 23.44 | 24.60 | 24.27 |
| GX160 | 25.76 | 26.03 | 23.65 | 24.19 | 25.35 |
| GX161 | 28.43 | 30.06 | 24.17 | 27.43 | 29.19 |
| GX162 | 22.85 | 24.12 | 21.83 | 24.37 | 21.94 |
| GX163 | 25.61 | 26.67 | 23.74 | 25.86 | 28.31 |
| GX164 | 25.33 | 23.93 | 23.59 | 20.78 | 25.63 |
| GX165 | 30.64 | 32.32 | 27.17 | 30.53 | 30.54 |
| GX166 | 25.26 | 25.63 | 23.06 | 23.79 | 24.97 |
| GX167 | 24.14 | 25.42 | 23.21 | 25.77 | 25.00 |
| GX168 | 22.42 | 25.13 | 21.26 | 26.69 | 24.69 |
| GX169 | 21.18 | 23.16 | 19.16 | 23.12 | 23.00 |
| GX170 | 21.59 | 22.42 | 20.00 | 21.66 | 22.92 |
| GX171 | 21.71 | 22.16 | 19.60 | 20.51 | 21.57 |
| GX172 | 21.91 | 22.54 | 20.42 | 21.69 | 21.23 |
| GX173 | 21.40 | 22.83 | 19.75 | 22.60 | 22.29 |
| GX174 | 22.62 | 24.10 | 21.23 | 24.18 | 23.31 |
| GX175 | 26.57 | 27.66 | 24.68 | 26.88 | 27.36 |
| GX176 | 26.24 | 28.03 | 24.39 | 27.95 | 26.75 |
| GX177 | 24.32 | 25.11 | 21.93 | 23.50 | 22.45 |
| GX178 | 34.38 | 35.10 | 31.26 | 32.69 | 33.04 |
| GX179 | 32.86 | 33.49 | 29.69 | 30.95 | 32.64 |
| GX180 | 26.61 | 27.84 | 26.06 | 28.51 | 27.91 |
| GX181 | 24.71 | 26.17 | 22.26 | 25.17 | 24.46 |
| GX182 | 24.82 | 24.62 | 24.09 | 23.68 | 26.32 |
| GX183 | 26.52 | 27.27 | 24.49 | 26.01 | 26.40 |
| GX184 | 25.80 | 26.39 | 23.73 | 24.91 | 27.15 |
| GX185 | 23.79 | 24.05 | 21.67 | 22.20 | 23.65 |
| GX186 | 23.86 | 24.45 | 22.58 | 23.75 | 24.83 |
| GX187 | 26.48 | 27.51 | 23.24 | 25.30 | 27.34 |
| GX188 | 23.03 | 24.04 | 21.37 | 23.38 | 24.31 |
| GX189 | 24.69 | 25.91 | 22.30 | 24.73 | 24.61 |
| GX190 | 25.99 | 26.72 | 24.38 | 25.85 | 26.21 |
| GX191 | 23.33 | 23.67 | 21.28 | 21.96 | 23.38 |
| GX192 | 27.24 | 28.51 | 25.83 | 28.38 | 29.11 |
| GX193 | 26.01 | 27.09 | 22.30 | 24.46 | 26.21 |
| GX194 | 25.85 | 26.92 | 23.30 | 25.44 | 26.75 |
| GX195 | 24.97 | 25.95 | 22.35 | 24.31 | 24.54 |
| GX196 | 23.02 | 24.12 | 21.82 | 24.01 | 22.86 |
| GX197 | 23.74 | 25.87 | 22.38 | 26.63 | 23.52 |
| GX198 | 25.00 | 25.55 | 22.34 | 23.45 | 26.32 |
| GX199 | 25.94 | 26.05 | 23.32 | 23.54 | 22.10 |
| GX200 | 21.41 | 20.74 | 20.66 | 19.33 | 20.47 |
| GX201 | 28.95 | 30.37 | 25.57 | 28.42 | 34.51 |
| K1561 | 31.94 | 32.99 | 29.58 | 33.68 | 32.18 |
| G1025 | 16.23 | 16.89 | 14.44 | 15.76 | 16.73 |
