# Supplementary material for: Identification of QTL TGW12 responsible for grain weight in rice based on recombinant inbred line population crossed by wild rice (Oryza minuta) introgression line K1561 and indica rice G1025
Source: BMC Genet. 2020 Feb 3;21:10. doi: 10.1186/s12863-020-0817-x (PMC6998338; doi:10.1186/s12863-020-0817-x)
Supplement: Supplementary file 2 — Additional file 2: Table S2. SSR primer sequences used in the present study. [file 12863_2020_817_MOESM2_ESM.docx]

Table S2 SSR markers used in present study

| SSR Markers | Forward Primer(5’to 3’) | Reverse Primer(5’to 3’) |
| --- | --- | --- |
| RM6296 | TTAAGCCCACGTTTCTCTTGTCC | CTCGCTAGGGTTAGGGTTTCAGG |
| RM27567 | CCAATAGCACCAAGGGCTTCC | GAGGCTATCCCTGCGTTAGAGG |
| RM27568 | TGTCATGTCGAGGATCGGAAGC | GAAACGATCTGGTTGCCTTCTCC |
| RM27569 | GAGGAGCAACACAAGAGAAGACG | CACACACTTGCCCTAAGATTCG |
| RM27573 | GCCTCAACAAGATTCGATTCACC | AAATCGGGTTCGGACTTATCAGG |
| RM27574 | CAAGTGCAACAGGATGACTGC | TATACGCGGAAAGCGATAAGAGG |
| RM27575 | TTTCCTTTCTTGCGCCATTTCC | GAACTCCTCCTGGCACAGAATAAACG |
| RM27576 | TAAGTGCATCCAGCAATTGACC | GCCGTCAGTTATAATCCTTCTTGG |
| RM27577 | AGTGGAAGCCCAAGCCAGAGG | CGTGACGTCTCCTTCCTTTCTCACC |
| RM27578 | TCTCTTCAGCTGGTGGTTCTTGC | GGAGTACGAGGACTGGATCAACG |
| RM27579 | TCCAACTCGAGGCTGGATAGC | CGTCGAGACGGAGGTTGAGC |
| RM27580 | TGATCGCCAGGATCTTGCTCTCC | GGGCTCTGCCTTATCGGATCG |
| RM27583 | ATGGTGTTGAAGGGCTTGTTGG | ATGCCCTGATAGGTGAACAGTCG |
| RM27584 | CTCTTCCTTCCTCCTCGCTTCG | GCTGCGGACGATTAGGATCTAGG |
| RM27585 | CCGCTCTCCCTCCTAGATCCTAATCG | GGTTGCTGGAGCCGAACATGG |
| RM27587 | CTCTGTGCCGATAAGGGATGAGG | CACATTGCTTACGAGGCTCTGC |
| RM27589 | CTATTTGGGTTTCCCACGATAGC | TTTCCCATCCTCTCAAACTCAGC |
| RM27590 | CTCGCTAGCCACCTGCTCAACC | TCCATCCCTTCTCCTCTCACATTCC |
| RM27591 | AGGCTTCTTCACGAACTAATGG | GGACAATCGGACAAGATGTACC |
| RM27593 | CGTGCTGTCTCGGTCTCTCTCC | CAGTTACAGGAGGGAATGGATGC |
| RM27594 | CCGGTCTTCAGCTTATGGAGTCATGG | CACCAGGGCATGCGTACACACC |
| RM27595 | GTTCATGTTCGACGAGCACACC | CTTCGCACACGAATCTCAAAGC |
| RM27596 | ATCCCGACCGTCCAGTTCAGC | GGTCTCGATCTCTTCGATGACTTGC |
| RM3472 | CACACACTCTCTCAATCTCAACACC | AGAAGCGAGAGGAGGGAGATAGC |
| RM27599 | GAGAGAAGGAACGGAAGGAAGG | CACCTGCTCTCCCAACTCTCC |
| RM27600 | ACCACCAGACGCCATCAAACC | CGATCCTCCTCCTCTCCTCTCC |
| RM27601 | GCTTAGATGGTGCACAGGAAACC | GCTGTGTTTGTTTGAAGGTGTTGG |
| RM27602 | GCACGTTCGCCGTCTTGTGG | GCGTTGGTGTGTGTTGTTCAATCC |
| RM27603 | GCACGTTCGCCGTCTTGTGG | GCGTTGGTGTGTGTTGTTCAATCC |
| RM27604 | AGGGAAGGAAGAAATGTGAAGC | CAAAGAATCCACCCAAGAAAGG |
| RM491 | CACATGATGCGTAGCGAGTTGC | TTATGCCTCTCCCTTCCCAATTCC |
| RM27607 | GACACCCGTGAACTTTGGATGG | TAGGGACGGAGGGAGTATGTGC |
| RM27608 | TGTTCAGTGGTGGTTCTGATTCC | CCACCAAAGATTAAGGGTGACTATGG |
| RM27609 | TGTTCAGTGGTGGTTCTGATTCC | CCACCAAAGATTAAGGGTGACTATGG |
| RM27610 | CAGGCTAGCGAATCCAAAGAGC | CCTTCGAGGACGAGAGTAAGTGG |
| RM27611 | TGGAGAGGGAGGATAATGAGTTTAGG | GTCGGCTTAAATACGCTGAGAGC |
| RM27613 | TCTTCGTGCTAGCTACTACCACTCG | CTCAACCGCATTGATTTCGTACC |
| RM27614 | AGACAAGTGGGAGCATAGGATGG | CTAGGATGTGTGCTTTCCTGTCACC |
| RM27615 | CCAGATCTGACCCAGCCACACC | GTCGCCGTGCCTAACCCTATCG |
| RM27617 | TCAGAGAAGGTGGTAGGTCCATCC | AAAGAGAGCCCAATCCCATCG |
| RM27618 | TGCATGGTCAACCATATGAAGC | TTTCACATCCCAGACGAGAGG |
| RM27625 | ATTGATCTAAACAGGCCCTAGC | GCAATGTCGCTGTACTTAGACC |
| RM27626 | GCACAAGACCACCACCTACCTACC | GAACTCGAAGCGAGGAGAGAGG |
| RM27627 | CCAACGATAGTTTCTAGCTAGGTTCC | TGGAGGTAATAGCTCATCAGTTGC |
| RM27628 | CGATCTCGCTCTGGTTGTGG | CTCTTGAGAGACGGAAACCAACG |
| RM27630 | CTCAACGCCCTTCCTCACTCG | ACTTGGCCCGAGCATCTACAGG |
| RM27632 | GAAGAAGCAGACAAGGAGAAGG | ATGGATCAAGGAGAGGATGG |
| RM27633 | AGAACCCTAAGCTGTCCCTCTCC | CGTGTACAAGTGGTTCTTCCTTTGC |
| RM27634 | GGACATCGCTAAATCAAGAACC | AGTATGTGTCTTCCACTCTTCTCC |
| RM27635 | CCAAACTATCAGCGACATGAACG | CTCTGTTGTTTGGGAGACTGTGC |
| RM27636 | GCACAGTCTCCCAAACAACAGAGG | CGTTGCTCTTGTTCTTGTTTCTGTCG |
| RM27637 | CGGTGGTGGCTTTCCAGAGG | TGGACGAGGACATGTCGATACAGG |
| RM27638 | AGATTCCCATCCGTTAGGAAAGC | GATGCACATGCACTTGTAGTTCC |
| RM27639 | GTCCTAGGCCGTCTTCTTTGTCC | CTAATGTCGTGTTGGGTTGTTCG |
| RM27640 | ATGTGCATTCTCCTCTCATCTGC | ATGGCAGGAATAGCAACAATCC |
| RM27641 | TCTTTCCTTCCTTCCTCCCTTTCG | AATACGTGGAGGACGTCATGTCTGG |
| RM27644 | CGGCAGCGCTAGCATCATCG | GTCACACTGCACACGGCGTAGC |
| RM27649 | AGGACAGGAGACACAAGGTTAGC | TGAAACGGAGGAAGTAACTACACC |
| RM27650 | AGCTTGACGTCATTGCTCTCAGC | GAGAGTACTGCTACCTCCGTTTCAGG |
| RM27651 | TGGAGTTGAGATCGACGTTGAGG | AGACCTTCCACGACGGCTTCC |
| RM27652 | TCCAAACCCACTGACCACTAAGC | CAATTGAACACGTACGCAGTGG |
| RM27653 | GTTGGTTGATTACCAGTTGGATGG | AATGTGTTGGCTCTTGAGTTGAGC |
| RM27654 | CGCGTACGTACTTCTTGGAATCG | ATGGGCGTCCTCTTCTTGTTGG |
| RM27658 | ATCATGGGACAGCACAACAGTGG | TTCAATTAGGCCACGCCTACTAACC |
| RM27659 | CGGCCAATATTTGCGAGTAAACG | ATCCACACTTCCACAGCAACACG |
| RM27663 | TAGCTAGGATCGGATGAAAGATCTCC | GGAGAGAATGTGCGTGCTTGC |
| RM27671 | CTGAGGTGGCACACTAATCTTCAGC | AGTGACCGAGTGCATGAGTACACC |
| RM27672 | CTGAGGTGGCACACTAATCTTCAGC | AGTGACCGAGTGCATGAGTACACC |
| RM27683 | TGAGTCGAGATTTCACATCAGG | TTTCCTTCCAAAGGTAGAGGTAGG |
| RM27684 | GACGCATCATCCAAATTCCAAAGG | CTTGCCACGTGTCGCTTAACTAAAGG |
| RM27685 | CCGACTTACATAGTGTGTGCCCTTGC | GGGACAACCGGACAATCTTCTCTGC |
| RM27686 | ATGGGAACAACCTTATCGTCTGC | GAGAGTTGGGCTTCTTGTTGAGG |
| RM27687 | ATGGGAACAACCTTATCGTCTGC | GAGAGTTGGGCTTCTTGTTGAGG |
| RM27688 | AAAGAAGGCACCTGTCATACTTGC | TTACGTGATATCGCTTGGATGG |
| RM27689 | AACCTGCAATTACCATCCAAGC | AATACACACCCACAGTTCCACACC |
| RM6998 | GATAAGCTTGCGGACCGGATTAAAGC | CTCCACGTTTGGGTCGCTACTCG |
| RM6606 | AAACCACCTCGTCGTCTTCTTCC | GCTGATGACGTTGAATGACTTGG |
| RM27694 | ACGTGCATTACGAGCAGACTGG | TGCGTGACTCTACCAAGTGTATGG |
| RM7619 | TCTTGGTATGTATTGGCAGCGAAAGC | AGGATGTGAATGAAGGCGAATGG |
| RM27696 | CTTGTCGTCATCTCAATCACAAGG | AAGTGACACATGAGAAGCCAACG |
| RM3455 | TGAATCCACACTCGCAGATCG | AAATCAGCTCGGAGGGAACAGC |
| RM27698 | TGCAACGGACCAGAATCCAATCC | CCGCAGTAACCACGCTATCAATCG |
| RM27699 | TGATGGACGTTTAAGTCGGATAGG | GAGGTCCAGGAAATTGATAGATGG |
| RM27700 | CGTTACTACGCGGTTCTGAGACG | TTGCCCTCTAATCACCATGTGC |
| RM27701 | CTATATGGCACCACTGACATTTGC | TCAATAAAGCCCACCGTTCC |
| RM27706 | ACTCCCTCCGACACCATCATCC | ATACGGGAACCCTCACGCTACC |
| RM27708 | CCCATCTCTACCCAATCACAAGG | GCGACACTCTCAGCTTCTACATCG |
| RM27709 | CGGCTCTATACCGGCAAGAATGG | CTCTGGTGCAAGGTGTCCAACC |
| RM5746 | CAGCTTCGGCAAAGCAAAGC | CTCGCTACGTCGACTGATTTGG |
| RM512 | TGCAGTGAATGGAGACCACTAGC | CGGTGAGTCCCATATCTTCAACC |
| RM27714 | AATCTTCTCGGTTTCGTGGAGAAGC | CATTGAGCTGGGCTGCAAAGG |
| RM27717 | CTGCACGTGCGTAATATTTGTGG | TGCATGACGATAGCTGAGAAGAGG |
| RM27719 | AACACCTAGCACGCAAGTACG | GTTACAAGGGAAATTCGTACCG |
| RM27722 | GATGCAACCACAACCACTTAGTAACC | TTCGCTTACTGATCGTCCTACAAAGC |
| RM27723 | GCCATGCCCTATATCAAAGTTGC | GCATAGAATTCGAAGCCATAGCC |
| RM27724 | CCTATGTGCACGTCCTTCATGC | GCAAACCAGAGAAGATCAGCAGAGG |
| RM27725 | GTACCGACAGGTGCGTACTAATCC | CAAGTAGGATGCCAACCATGTCC |
| RM27726 | ATGCCACTCCAACATGTAGCC | AGGAGCGTTCGTAGGAAGTAGC |
| RM27727 | AACCAGCTGTCGATTTATGTCAGG | GAGATTGGTCGATGTCCAGAAGG |
| RM27730 | CCAGTTAATTGGGTTGGTTCATGG | CGGGACGAATAATAGATGGATGG |
| RM27731 | CACTGTAGAGAGCTTCCATTGC | GCTATAGTCACTCACTCTCTCACACC |
| RM27735 | ATCGGTTCTTCCCTTCTTCTTGC | TTTGCTTATACCGCTTCCGATCC |
| RM27746 | CGCTACTGCATCATCTGTACTACTGC | TCTATTTCTATCACGTGGGCTTGG |
| RM27747 | CGCTACTGCATCATCTGTACTACTGC | TCTATTTCTATCACGTGGGCTTGG |
| RM27749 | AGAGCTCAGCGCCTAAGTTATTGG | ACGTATGGGTGTCTCTCGTCAAGG |
| RM27757 | ACTGCGTCCGCAGATAGGGTAGG | CTGGCTCTGTTGATAAAGGGAGTGC |
| RM27760 | GAATGGTGATGCGTGTAATGATGG | GGTTTAGATTGTGGATGCATGTGAGC |
| RM27767 | CCCTAATTTCGGTGTGATTGG | ACGAAGATTGTGGAGGACTGC |
| RM27774 | CGTCCTTGACAAGTTGACATCC | TTCTTCTTCTCTCTTGTCCCTAGCC |
| RM27777 | CATCAACCCATCCTACGTTCTACG | ATGGTTATTGTCCGCCGAAGC |
| RM7119 | CTGAGACCATGACGGGATAAACACC | GGCCTCAGATCATCACAACTTGG |
| RM27781 | CACGGTGTTCAACTACGGTCTGC | ATCATGTGGTCCAATCAACTGTGG |
| RM27782 | GAGGAGAGGAGACGGAGAGG | CGAGAGTGGTGATCTCACTTAATAGG |
| RM27783 | CCTTGAACAAGTGACCTACACAATGC | TCCAAACGAAACACCCTCAACC |
| RM7003 | CTCTAGCTCTCTCATGGATGG | AATCATAGGGCAGACATACAGC |
| RM27571 | TGTAGCTTGCACGAATCTTAGGG | CGAATCTTGGAACACATCAACG |
| RM27586 | CCTCCGTACTCGTAAAGGAAGTCG | TCTCCGTACCAGTAAAGGAAGTCG |
| RM27592 | GCAAAGCAAAGGGAAAGAAGATGG | GGTCAGACCAGCGTTGAGTGG |
| RM7448 | GACTTTGGCGGATTGATTGC | GCTGGCTAATAGTGTGCTATTGTACC |
| RM27612 | GAGCTAGAGCTGTGCCAAACAGG | GAACCCTTCAATTGTGCCATAGG |
| RM27616 | TGATATTGGGCCTTCAGTTGTGG | TGACCAACGTTTGACCATTCG |
| RM27622 | CTCTTCTTTCGCCAGCCTCTCC | GGGACGGTTTGAAGGCAAGC |
| RM27623 | TAGGGAACGTGAACGTGAAATGC | CTAGGGCTAGCTCTTCCACTCAGG |
| RM27624 | AACACAATTCACCGGTCCTTAGC | GATCGATCTTTAGTCCCGGTTGG |
| RM27642 | CTACTACAAATGTCCTTCCGAACC | TGGCTTCAACATAAGCAAGG |
| RM27643 | TTGCCGTGCATGAAGTACTCTGG | CGGCAAATTCGGGTTTGTAGC |
| RM27645 | GTCCCTCAAGCCGAAGTGGTAGC | ATCGCGATCAGCCATCTGTGC |
| RM27646 | CGTCGAAGTCGAAGTCGTAGTATAGC | TTACCAACCGGGACTAAAGATCG |
| RM27647 | CGTCGAAGTCGAAGTCGTAGTATAGC | TTACCAACCGGGACTAAAGATCG |
| RM27648 | CGTCGAAGTCGAAGTCGTAGTATAGC | TTACCAACCGGGACTAAAGATCG |
| RM27655 | TTCTCTCCGATGCTGATGTTACC | CGGATTGGTCCACAAGAAAGG |
| RM27668 | ATGCAAATGTGGGAGAACATGC | GACGACTTGAGAGCGTTTGTAGG |
| RM27669 | ATGCAAATGTGGGAGAACATGC | GACGACTTGAGAGCGTTTGTAGG |
| RM27670 | ATGCAAATGTGGGAGAACATGC | GACGACTTGAGAGCGTTTGTAGG |
| RM27690 | CCCAAAGGATTGTGAGAGACACC | TCATCCAGATTCCAGAGGTTTCC |
| RM27693 | TGGGCAGATGGACGTGTTACC | CTTATGGACTCGGATGATTTGTCG |
| RM27715 | TAGAAGAAACCGGAGGGAAGTGC | TGGGTTAGGTTAGGTGGGATGG |
| RM27716 | TTAGCAAGGTGTTGCTTACC | GAGCAGTACTAGCATTGTTGG |
| RM27718 | AAATCAAAGGAGGGAGATCG | ATAGAGGGCTTGGTTTGTTCC |
| RM27720 | GAGTTGGATACAAACGCATGG | GTTACAAGGGAAATTCGTACCG |
| RM27721 | GAGTTGGATACAAACGCATGG | GTTACAAGGGAAATTCGTACCG |
| RM4173 | CCTCCATACTCACAAAGGAAGTCG | TCCCTCCGTACTTATAAAGGAAGTCG |
| RM4173 | CCTCCATACTCACAAAGGAAGTCG | TCCCTCCGTACTTATAAAGGAAGTCG |
| RM27732 | CTGCGACATCTGCCAGGTACG | GGCAATCATCTAAATGCCTGAGTACG |
| RM27733 | TGTCAGAGTTGCTTGTTACG | GGATTTCTAGTCCCTGTTGG |
| RM27734 | GGACGTCATCGGACCACTCG | GAGAAACTTACCGCCGAGTTTGG |
| RM27742 | AATTCGTAACGTCGGTCCTTCC | TTATTTGCATGGCCCTGTTTCC |
| RM27743 | AATTCGTAACGTCGGTCCTTCC | TTATTTGCATGGCCCTGTTTCC |
| RM27744 | TTATTTGCATGGCCCTGTTTGC | CGTCGGTCCTTCCTCTTCTAACG |
| RM27748 | AATCCATAACGTCGGTCCTTCC | TATTTGCATGGCCCTGTTTGC |
| RM27750 | GATCGATCTTTAGTCCCGGTTGG | AACACAATTCACCGGTCCTTAGC |
| RM27751 | TAGGGCTAGCTCTTCCACTCAGG | ATGAGAACGAGCGAACGAACG |
| RM27752 | AATTCGTAACGTCGGTCCTTCC | AACACAACCGGGACTAAAGATCG |
| RM27753 | AATTCGTAACGTCGGTCCTTCC | AACACAACCGGGACTAAAGATCG |
| RM27756 | ATCATGGCATAAGCAGTGTAGGG | AAAGATCAGCTCTGACCCTATTCC |
| RM27758 | CGACGTCAAACATTTAGGGAAGG | GCAACGATAACAGCAGACAAAGC |
| RM27759 | CGACGTCAAACATTTAGGGAAGG | GCAACGATAACAGCAGACAAAGC |
| RM27761 | GTAGATTGTGGGTGCATGTGTGC | TGAGAGGTATCCATCCGTGTTAGG |
| RM27762 | TGAGACGATCGAACTCCTAACTGC | TCGGACTCGACTCCTATGAAGG |
| RM27766 | ATGGTGCCCGAGTACAATGG | TGAGGTGCATGTGAACCTTGG |
| RM27771 | GCTCAATTAGCCCAATTACTCG | TACGTACACGGGAAGGAGTAACC |
| RM27772 | GCTCAATTAGCCCAATTACTCG | TACGTACACGGGAAGGAGTAACC |
| RM27773 | GCTCAATTAGCCCAATTACTCG | TACGTACACGGGAAGGAGTAACC |
| RM27775 | GCACCACCTCCACCATCTCC | TTGCACGACTGCTCGTTCTCG |
| RM27776 | GAACGTGTACGCGCTGTTGC | GCTCCCTGTTGACTGGTTGAGG |
| RM27779 | TACCAATCCGGGACTAAAGATCG | CTACGACGGAAACACAATTCACC |
| RM3917 | ACTCAGGCTTGACGGACGACTCC | CGAACGAACGAGGACGAACG |
