# Supplementary figures and images for: Identification of QTL TGW12 responsible for grain weight in rice based on recombinant inbred line population crossed by wild rice (Oryza minuta) introgression line K1561 and indica rice G1025
Source: BMC Genet. 2020 Feb 3;21:10. doi: 10.1186/s12863-020-0817-x (PMC6998338; doi:10.1186/s12863-020-0817-x)

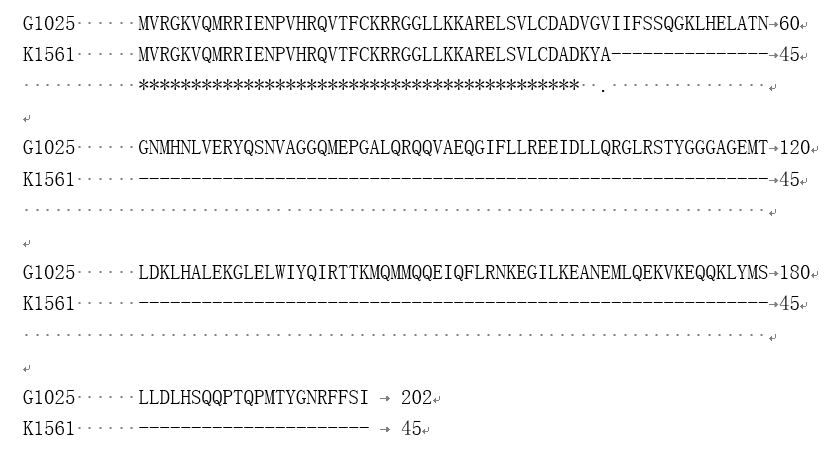

Supplement: Supplementary file 3 — Additional file 3: Figure S1. Alignment of amino acid sequences of ORF12 between G1025 and K1561. [file 12863_2020_817_MOESM3_ESM.jpg]
